# Evolution of* bopA* Gene in* Burkholderia*: A Case of Convergent Evolution as a Mechanism for Bacterial Autophagy Evasion

**DOI:** 10.1155/2016/6745028

**Published:** 2016-11-27

**Authors:** Dong Yu, Zhiqiu Yin, Yuan Jin, Jing Zhou, Hongguang Ren, Mingda Hu, Beiping Li, Wei Zhou, Long Liang, Junjie Yue

**Affiliations:** ^1^Beijing Institute of Biotechnology, Beijing, China; ^2^The Second Military Medical University, Shanghai, China; ^3^Anhui University, Hefei, Anhui, China

## Abstract

Autophagy is an important defense mechanism targeting intracellular bacteria to restrict their survival and growth. On the other hand, several intracellular pathogens have developed an antiautophagy mechanism to facilitate their own replication or intracellular survival. Up to now, no information about the origin or evolution of the antiautophagic genes in bacteria is available. BopA is an effector protein secreted by* Burkholderia pseudomallei* via the type three secretion system, and it has been shown to play a pivotal role in their escape from autophagy.  The evolutionary origin of* bopA* was examined in this work. Sequence similarity searches for BopA showed that no homolog of BopA was detected in eukaryotes. However, eukaryotic linear motifs were detected in BopA. The phylogenetic tree of the BopA proteins in our analysis is congruent with the species phylogeny derived from housekeeping genes. Moreover, there was no obvious difference in GC content values of* bopA* gene and their respective genomes. Integrated information on the taxonomic distribution, phylogenetic relationships, and GC content of the* bopA* gene of* Burkholderia* revealed that this gene was acquired via convergent evolution, not from eukaryotic host through horizontal gene transfer (HGT) event. This work has, for the first time, characterized the evolutionary mechanism of bacterial evasion of autophagy. The results of this study clearly demonstrated the role of convergent evolution in the evolution of how bacteria evade autophagy.

## 1. Introduction

Autophagy is an intracellular degradative process that maintains cellular homeostasis and acts as a cell quality control mechanism to eliminate aged organelles and unnecessary structures [1]. As a cell-autonomous, innate immune response, autophagy has been demonstrated to be an important defense mechanism targeting intracellular bacteria to restrict their survival and growth [2]. It has been reported that some intracellular bacteria are targeted by the autophagic system for lysosomal fusion and degradation. The first example of autophagy targeting intracellular pathogenic bacteria was that of ΔactA-mutant* L. monocytogenes*. In 2003, Rich et al. observed that ΔactA-mutant* L. monocytogenes* was captured in autophagosomes [3]. Since that study, extensive works have been done on antibacterial autophagy to determine the induction and targeting mechanisms of this process.

Bacterial autophagy has been highlighted as a fundamental host cell response to bacterial invasion. Notably, there is increasing evidence to suggest that pathogenic bacteria have evolved many strategies to combat the host autophagy machinery [4]. Certain bacteria can directly interact with the host autophagy signaling and/or autophagy proteins to inhibit the signaling pathways that lead to autophagy induction, use virulence factors to camouflage them to avoid autophagic recognition, or antagonise autophagy initiation or autophagosomal maturation [5]. Some bacteria even actively exploit components of the autophagy pathway to facilitate their own replication or intracellular survival [4].

Understanding how novel functions arise is an intriguing question in many fields of biology. However, no information about the origin of how intracellular pathogenic bacteria block or hijack the autophagic process is available.

It is believed that bacteria escape autophagy by using their effector proteins to mimic the functions of host autophagy components [6]. Currently, two theories have been proposed to explain how bacterial pathogens acquire their eukaryotic-like proteins: convergent evolution and horizontal gene transfer (HGT). In the former, pathogen-encoded factors independently evolve features of host components, presumably via random mutations and natural selection. In the latter, the pathogen acquires host genetic material via horizontal gene transfer, followed by selection to increase the pathogen's fitness. Such events of gene transfer from eukaryotes to bacterial pathogens may occur during early evolutionary time or in recent evolutionary time [7].

In this work, we focus on the evolutionary origin of* bopA* in* Burkholderia pseudomallei* to characterize the evolutionary mechanism of evading autophagy by bacteria.* B. pseudomallei* is an intracellular pathogen that causes melioidosis. Cullinane et al. found that the pathogen can actively evade autophagy by protein BopA, an effector protein of* B. pseudomallei* type III secreted system (T3SS) [8]. Using sequence similarity searches, motif detecting, and statistical comparisons of both GC content and codon adaptation index (CAI) in conjunction with positive selection analysis, the results of this study suggest the* bopA* gene does not arise from HGT from the host cells but rather represents a case of convergent evolution.

This is to our knowledge the first report to explore the evolution of autophagy-evading function in bacteria. A better understanding of the mechanisms by which bacteria manipulate autophagy will inform the therapeutic treatment of bacterial infections [6]. Our results will provide new insights into the mechanisms of pathogen-host autophagy interactions.

## 2. Results

### 2.1. Sequence Similarity Searches for BopA in Eukaryotes

Recent studies have shown LC3 is recruited to* B. pseudomallei*-containing phagosomes [8]. Mutants lacking the BopA resulted in delayed or no escape from phagosomes. The BopA is required for* B. pseudomallei* to evade the host autophagy defense system [8]. To investigate the homologs of BopA, NCBI-BLAST searches were conducted versus the NCBI nr protein sequence database with a cutoff *e*-value of 1*e* − 3 by using the BopA protein sequence as the query sequence. No homologs of BopA were identified in any eukaryotic organism.

To investigate if remote homologs of BopA exist in eukaryotes, we inspected BLAST hits above the default e-value cutoff. No related sequence was found in any eukaryotes, even at an *e*-value of 1.0.

**Figure 1:**
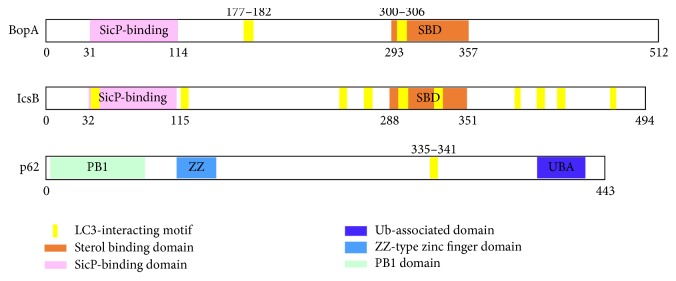
Graphic representation of the sequence domain and motif in BopA, IcsB, and p62. Yellow shading denotes LIR motif; LIR named LC3-interacting region mediates binding to all the members of the mammalian ATG8 family of proteins.

It has been reported that two domains might be remotely related to BopA (Figure 1). The first is a SicP binding domain at the N-terminus [9]. The second is a cholesterol binding domain (SBD) [10]. To investigate whether these two domains are found in eukaryotic proteins, we conducted a further PSI-BLAST search using the protein sequences. As a result, the two domains of BopA were also not present in any eukaryotic protein sequence.

It has been observed that bacterial pathogens can obtain virulence factors by horizontal acquisition of eukaryotic genetic material [11–14]. Events of gene transfer occurring in recent evolutionary time are likely to lead to significant sequence similarity between the pathogen protein and the host protein [7]. Recent HGT events can be easily identified by looking for high-scoring sequence matches in eukaryotic host.

In our results, no homologs of BopA and its domains were detected in any eukaryotic genome. These results indicted the* bopA* gene was not acquired from the eukaryotes through a recent HGT event.

### 2.2. BopA Gene in Bacteria

BopA displays no sequence similarity to any known eukaryotic protein either at the domain level or to the full-length protein. This indicates that the origin of* bopA* gene was not the result of a recent HGT event from eukaryotes. However, HGT events of gene transfer from eukaryotes to bacterial pathogens may occur during early evolutionary time; anciently transferred genes may have a much broader presence across multiple species and may be diverged from their homolog in the donor genome.

BLAST searches against NCBI nr databases revealed that close homologs of BopA were only identified in* Burkholderia* species with >90% identity and 1*e* − 10  *e* value (*B. pseudomallei*,* B. mallei*, and* B. thailandensis*), and BopA exhibits 23% identity with IcsB of* Shigella*, which has been characterized as a virulence factor playing an important role in helping* Shigella* to escape autophagy [15]. The* Shigella* IcsB protein interferes with autophagy systems by binding to the* Shigella* surface protein IcsA, thereby competitively inhibiting binding of the autophagy protein Atg5 to IcsA. Although the mechanism(s) by which* B. pseudomallei* evade autophagy remains unknown, it is unlikely that BopA acts in a manner analogous to IcsB which facilitates bacterial evasion of autophagy [16, 17]. When the sequence identity falls within or below the twilight zone of 20–25%, the evolutionary relatedness of proteins cannot be assumed [18]. Based on the above observations, the evolutionary relationship between* Shigella* IcsB and BopA is uncertain.

**Figure 2:**
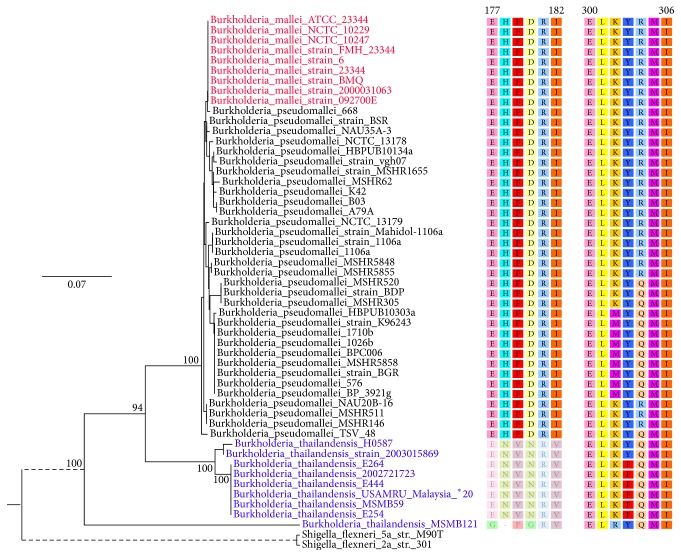
Neighbor-joining tree of the BopA protein in all 51 completely sequenced* Burkholderia* strains (left). Two Shigella_flexneriIcsB were selected as the outgroup to root the tree. The numbers at each node were the bootstrap values. The two LIR motifs are marked on the right side of the phylogenetic tree (right). LIR motif at 177–182 from BopA of 9 B. thailandensis strains is absent.

Our phylogenetic analysis of BopA sequences reveals that the* B. mallei* BopA clade is more closely related to* B. pseudomallei* than to* B. thailandensis* (Figure 2). This result is in agreement with tree topology derived from 16S rRNA,* recA*,* gyrB*,* rpoB*, and* acdS* gene sequences by Paulina et al. [19].

Anciently transferred genes that were acquired before speciation events may have a much broader presence across multiple species [7]. Information on the taxonomic distribution and phylogenetic relationships of the BopA protein revealed that the* Burkholderia bopA* gene might be not acquired from eukaryotes through an ancient horizontal gene transfer (HGT) event.

Next, we investigated the prevalence of the* bopA* gene in* Burkholderia* strains by genome sequence analysis. The 225 full or partial* Burkholderia* genome sequences, including 184* B. pseudomallei*, 25* B. thailandensis*, and 16* B. mallei* strains, were interrogated using the K96243 BopA protein sequence (accession number YP_111530.1) using tBLASTn to determine prevalence. Of the above 225 genomes, 174 (94.6%)* B. pseudomallei*, 23 (92%)* B. thailandensis*, and all 16* B. mallei* strains harbored BopA with 99% amino acid sequence identity to the BopA of* B. pseudomallei* strain K96243. BopA was present almost universally in the analyzed* Burkholderia* strains, suggesting that the* bopA* gene may have emerged in* Burkholderia*. These data suggest that it may have emerged very early in the* Burkholderia* lineage, nearly at the time of divergence from related genera.

### 2.3. The Nucleotide Composition of* bopA* Compared to the Whole Genome

Nucleotide composition is found to be variable among species, caused by variation in selection, mutation bias, and biased recombination associated DNA repair [20]. The comparison of GC content of a gene and its corresponding genome provides an important insight into the phylogeny of this gene. GC content of newly acquired gene is found to differ from GC content of whole genome. This provides an important conclusion in finding whether the gene is transferred horizontally. If a putative gene is an ancestral gene, it should retain a similar nucleotide composition as the rest of the genome. Similarly, exogenous gene by HGT could be detected by a significant difference of nucleotide composition to whole genome.

To confirm whether* bopA* gene was an ancestral gene, we used CodonW software computing G+C content of* bopA* and whole genome. The analysis of GC content fails to support the idea of HGT of* bopA* gene. The difference in the GC content of* bopA* gene and whole genome is little for all 51 completely sequenced* Burkholderia* strains. The G+C content of* bopA* and genome are 69.0% ± 0.003 and 68.4% ± 0.014 (Table S1 in Supplementary Material available online at http://dx.doi.org/10.1155/2016/6745028). Similar observation can be seen in the codon adaptation index (CAI) values [21]. The CAI values of* bopA* and genome are 0.247 ± 0.003 and 0.189 ± 0.002. BopA gene in all 51 completely sequenced* Burkholderia* strains is also found to be well adapted to their respective genomes as depicted by CAI. These results further raise the possibility that the* bopA* is an endogenous gene in* Burkholderia*, not horizontally transferred from eukaryotic host.

### 2.4. The Detection of Autophagy-Related Motif in BopA

The mechanism by which BopA can block or hijack autophagy is still unknown. It is reported that ActA of* Listeria monocytogenes* employs molecular mimicry by the WH2 motif to overcome autophagy and initiate bacterial motility [22, 23].* Listeria* ActA has two WH2 motifs that bind to Arp2/3 complex. The WASP-homology 2 (WH2) motif is an necessary element for the regulation of the actin assembly and Arp2/3 complex recruitment in mammalian Wiskott-Aldrich syndrome protein (WASP) family. To examine whether eukaryotic linear motifs exist in BopA, we performed a motif scan on BopA sequences using the search server of the Eukaryotic Linear Motif database.

**Figure 3:**
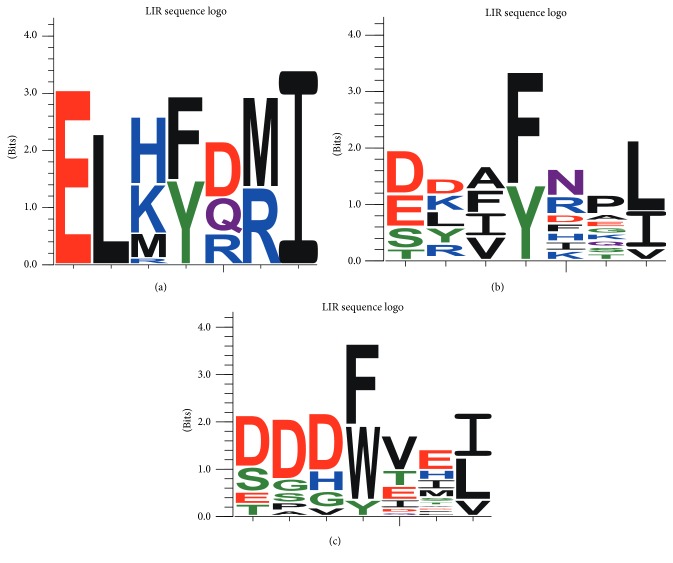
The consensus sequence logo for the LIR motif. The three logos are based on different LIR motifs distributed in the protein BopA of* Burkholderia* (a), the protein IcsB of Shigella, and 21 different p62 proteins that all bind directly to ATG8 family proteins in human (c). The pattern of LIR motif is** D/S/E/TX**{0, 2}**F/W/YXXI/L/V**.

Two copies of the autophagy-related LC3-interacting region (LIR) motif were detected in BopA of* B. pseudomallei* and* B. mallei* strains, which are located at residues 177–182 and 300–306 with 5.200*e* − 03 of Motif Probability Cutoff (*P* < 0.01) (Figures 1 and 3). IcsB protein was also detected to contain the LIR motifs. It is noteworthy that the LIR motif of 177–182 was lacking in BopA homologs found in the closely related* B. thailandensis*, which is avirulent (Figure 2).

LIR motif mediates binding to ATG8/LC3/GABARAP and is required for autophagic degradation of p62 [24–26]. Pankiv et al. and Ichimura et al. have independently showed that the mammalian autophagic cargo receptors p62 and other ATG8-interactors bind directly to LC3 via the LIR motif [25, 26]. BopA has previously been showed to inhibit recruitment of LC3 to pathogen-associated phagosomal structures during evasion of autophagy [27]. BopA is likely to use LIR motif to inhibit recruitment of LC3 to successfully avoid autophagy.

Short linear motifs (SLiMs) are evolutionary plastic modules and unstructured elements of proteins. SLiMs can facilitate adding novel protein-protein interaction interfaces and provide compact interaction interfaces [27] and are ubiquitously used in interactions between pathogens and their hosts [28, 29]. It has been suggested that pathogens may employ eukaryotic linear motifs to block or hijack the host cellular machinery during infection [30]. Due to the small number of mutations necessary for the formation of a novel motif, SLiMs are considered to emerge in pathogens in a convergent manner.

The fact that BopA contains eukaryotic autophagy-related motifs but has no homolog in eukaryotes suggests* bopA* may be the product of convergent evolution. For evading autophagy, the LIR motifs in BopA are likely to be formed by convergent evolution via random mutations and selection pressure to mimic the function of p62 binding to LC3. This is consistent with the fact that the LIR motif at 177–182 is absent from BopA of* B. thailandensis*, which exists in the soil in Thailand but is avirulent and rarely causes disease [31]. The absence of LIR motif at 177–182 from BopA of* B. thailandensis* may be due to a relatively weak selection pressure from the host autophagy system.

### 2.5. Positive Selection Analysis of BopA

The evolutionary distance between human and bacteria results in clear differences in structure and function between their proteins. To interfere with the host pathways successfully, bacterial virulence factors have to solve the contradiction between the distant evolutionary relations, which implies that the functional similarity with host proteins is needed for the virulence function of bacterial factors. It can be imagined that convergent, function-driven evolution would be under selective pressure [32]. For pathogenesis to occur, bacterial virulence factors may be under selective pressure to mimic components of their host's metabolic or immune system pathways.

It was reported that a number of virulence genes, especially associated with evasion of host immune defense, were under a strong adaptive selection pressure [33]. In a previous work, Karn and Laukaitis have demonstrated that two rodent kallikrein subfamilies, which had experienced convergent evolution, also have apparently evolved under the influence of positive selection [34]. Genes involved in adaption and functional innovation often show the footprints of positive selection [34, 35].

Considering the key role of BopA in the evasion of autophagy from the host, it was particularly interesting to determine whether* bopA* was undergoing selection pressure. Thus, the codeml program of PAML [28] was employed to carry out the analysis of positive selection. Based on the LRT statistic for comparing the null model M7 and selection model M8 with *χ* ^2^ distribution, BopA was identified to be under strong positive selection with a significant *p* value 0.0003. In addition, two positively selected sites were determined: 5(G), 172(R). This result further raises the possibility of the convergent evolution of* bopA*.

## 3. Discussion

The survival of intracellular pathogens largely depends on their ability to influence and modulate defence pathways in eukaryotic host cells. Pathogens have developed a wide variety of mechanisms to subvert host-cell signaling pathways and the immune response. These mechanisms were thought to be attained mainly by pathogen-encoded “virulence factors.” Several pathogenic microorganisms are known to produce proteins that mimic the form and functions of host proteins to exploit cellular machinery and counter immune defences.

As an important defense mechanism, autophagy can be used by eukaryotic cells to defend against microbes. A variety of host mechanisms exist for recognizing and targeting intracellular bacteria to autophagosomes. However, several intracellular bacteria have evolved ways to manipulate, inhibit, or avoid autophagy in order to survive in the cell. It remains unclear how pathogens have evolved specific mechanisms to manipulate autophagy [6].

In this work, the evolution of gene* bopA* of* B. pseudomallei* was investigated. With an aim to seek its origin, we conducted a search for homologs and analogs of BopA in publicly available protein sequence databases. Our approaches failed to identify any BopA homologs in eukaryotes, they indicated that the* bopA* genes are not likely acquired recently from the eukaryotic host via horizontal gene transfer. We just detect the homologs of* bopA* in* Burkholderia* species suggesting that* bopA* is not ancient horizontal transferred gene from eukaryotes. The analysis of GC content and CAI values further abandons the idea of HGT of this gene. The difference in the GC content of* bopA* gene and whole genome is little for all* Burkholderia* isolates. However, we provide evidence that BopA possesses eukaryotic linear motifs. These results support the hypothesis of an emergence of* bopA* by convergent evolution. Our researches provide probability action site of BopA binding to LC3-associated autophagy for subsequent experimental work analyzing evasion of autophagy.

Convergent evolution of bacterial virulence factors toward host components is not a novel idea [36]. The functional or active-site convergent evolution has been demonstrated for several virulence factors. This paper, for the first time, shows the evolutionary mechanism of autophagy evasion by bacteria and elucidates the role of convergent evolution in the function of bacterial antiautophagy gene. Our results bring new insights into how bacteria evolved the ability to evade autophagy and improve our understanding on the interaction between pathogens and autophagy.

## 4. Materials and Methods

### 4.1. Sequence Analysis of BopA

All genomic sequences of* Burkholderia* strains and BopA gene sequences were downloaded from the NCBI database. Then a homologous search of BopA was conducted using the K96243 BopA protein sequence (accession number YP_111530.1N) against the NCBI nr protein sequence database using NCBI-BLAST online version. Meanwhile, PSI-BLAST was employed to the domain searching using the domain sequences of BopA as queries. The multiple sequence alignment was implemented by Muscle.

### 4.2. Prediction of Motifs

The motif prediction was conducted using online Eukaryotic Linear Motif database (http://elm.eu.org). All the predicted motifs were shown in logo that was generated by WebLogo 3 with default parameters (http://weblogo.berkeley.edu/logo.cgi).

### 4.3. GC Content and Codon Bias

GC content and codon adaptation index (CAI) of* bopA* gene or all the genes in genome were both calculated using the software CodonW (https://sourceforge.net/projects/codonw/).

### 4.4. Phylogenetic Analysis

Prior to phylogenetic analysis, all the* bopA* gene sequences were aligned via the software MEGA4. The software MEGA4 was also used to construct phylogenetic trees by the neighbor-joining method. The numbers at node represent bootstrap values (based on 100 resampling tries).

## Supplementary Material

The GC and CAI values for the gene BopA and the genome that BopA locates, respectively.

## Data Access

All genomic sequences of* Burkholderia* strains and* bopA* gene sequences were downloaded from the NCBI database accession numbers of* Burkholderia* genomic sequences and* bopA* gene sequences can be found in the supporting file 2. Accession numbers of IcsB of* Shigella flexneri5a* str. M90T and* Shigella flexneri2a* str. 301 are NC_002698.1 and NC_004851.1, respectively.
