# Supplementary material for: Evolution of bopA Gene in Burkholderia: A Case of Convergent Evolution as a Mechanism for Bacterial Autophagy Evasion
Source: Biomed Res Int. 2016 Nov 27;2016:6745028. doi: 10.1155/2016/6745028 (PMC5149610; doi:10.1155/2016/6745028)
Supplement: Supplementary file 1 — The GC and CAI values for the gene BopA and the genome that BopA locates, respectively. [file 6745028.f1.zip › mat.6745028.v1.docx]

|  | %GC Value | | CAI value | |
| --- | --- | --- | --- | --- |
|  | Genome | Gene | Genome | Gene |
| Burkholderia mallei ATCC 23344 | 65.3 | 69.5 | 0.191 | 0.246 |
| Burkholderia mallei NCTC 10229 | 65.0 | 69.5 | 0.192 | 0.246 |
| Burkholderia mallei NCTC 10247 | 65.0 | 69.5 | 0.192 | 0.246 |
| Burkholderia mallei strain FMH 23344 | 65.3 | 69.5 | 0.191 | 0.246 |
| Burkholderia mallei strain 6 | 65.1 | 69.4 | 0.191 | 0.246 |
| Burkholderia mallei strain 23344 | 65.6 | 69.5 | 0.190 | 0.246 |
| Burkholderia mallei strain BMQ | 65.3 | 69.5 | 0.193 | 0.246 |
| Burkholderia mallei strain 2000031063 | 65.3 | 69.5 | 0.193 | 0.246 |
| Burkholderia mallei strain 092700E | 65.4 | 69.5 | 0.191 | 0.246 |
| Burkholderia pseudomallei strain K96243 | 69.3 | 69.0 | 0.188 | 0.247 |
| Burkholderia pseudomallei 1710b | 69.2 | 69.0 | 0.189 | 0.247 |
| Burkholderia pseudomallei 668 | 69.3 | 69.1 | 0.188 | 0.247 |
| Burkholderia pseudomallei 1106a | 69.4 | 69.2 | 0.186 | 0.245 |
| Burkholderia pseudomallei 1026b | 69.3 | 69.0 | 0.188 | 0.247 |
| Burkholderia pseudomallei BPC006 | 69.3 | 69.0 | 0.186 | 0.247 |
| Burkholderia pseudomallei MSHR305 | 68.7 | 68.5 | 0.188 | 0.248 |
| Burkholderia pseudomallei NCTC 13179 | 68.9 | 68.7 | 0.187 | 0.247 |
| Burkholderia pseudomallei NCTC 13178 | 68.9 | 68.6 | 0.187 | 0.250 |
| Burkholderia pseudomallei NAU20B-16 | 69.1 | 68.9 | 0.186 | 0.246 |
| Burkholderia pseudomallei MSHR511 | 69.1 | 68.9 | 0.186 | 0.246 |
| Burkholderia pseudomallei MSHR146 | 69.1 | 68.9 | 0.187 | 0.246 |
| Burkholderia pseudomallei MSHR520 | 68.6 | 68.5 | 0.185 | 0.248 |
| Burkholderia pseudomallei strain BDP | 68.7 | 68.5 | 0.186 | 0.248 |
| Burkholderia pseudomallei strain BSR | 69.2 | 69.0 | 0.187 | 0.247 |
| Burkholderia pseudomallei HBPUB10303a | 69.2 | 69.0 | 0.186 | 0.246 |
| Burkholderia pseudomallei HBPUB10134a | 69.2 | 69.0 | 0.187 | 0.246 |
| Burkholderia pseudomallei MSHR5858 | 69.3 | 69.0 | 0.187 | 0.247 |
| Burkholderia pseudomallei MSHR5848 | 69.1 | 68.8 | 0.185 | 0.245 |
| Burkholderia pseudomallei MSHR5855 | 69.0 | 68.8 | 0.188 | 0.245 |
| Burkholderia pseudomallei strain BGR | 69.3 | 69.0 | 0.187 | 0.247 |
| Burkholderia pseudomallei strain Mahidol-1106a | 69.3 | 69.2 | 0.185 | 0.245 |
| Burkholderia pseudomallei strain 1106a | 69.3 | 69.2 | 0.187 | 0.245 |
| Burkholderia pseudomallei strain MSHR1655 | 68.9 | 68.7 | 0.186 | 0.247 |
| Burkholderia pseudomallei 576 | 69.0 | 68.8 | 0.187 | 0.247 |
| Burkholderia pseudomallei NAU35A-3 | 69.0 | 68.9 | 0.185 | 0.247 |
| Burkholderia pseudomallei MSHR62 | 69.3 | 69.0 | 0.187 | 0.246 |
| Burkholderia pseudomallei B03 | 69.2 | 68.8 | 0.185 | 0.246 |
| Burkholderia pseudomallei TSV 48 | 69.1 | 68.9 | 0.187 | 0.247 |
| Burkholderia pseudomallei K42 | 69.2 | 68.8 | 0.187 | 0.247 |
| Burkholderia pseudomallei A79A | 69.1 | 68.8 | 0.187 | 0.246 |
| Burkholderia pseudomallei BP_3921g | 69.5 | 69.1 | 0.187 | 0.247 |
| Burkholderia pseudomallei strain vgh07 | 69.4 | 69.1 | 0.186 | 0.245 |
| Burkholderia thailandensis E264 | 68.4 | 68.6 | 0.186 | 0.256 |
| Burkholderia thailandensis MSMB121 | 69.1 | 68.6 | 0.188 | 0.259 |
| Burkholderia thailandensis H0587 | 69.0 | 68.8 | 0.187 | 0.250 |
| Burkholderia thailandensis 2002721723 | 68.5 | 68.6 | 0.186 | 0.256 |
| Burkholderia thailandensis E444 | 68.8 | 68.7 | 0.187 | 0.255 |
| Burkholderia thailandensis USAMRU Malaysia #20 | 69.0 | 68.8 | 0.185 | 0.255 |
| Burkholderia thailandensis MSMB59 | 68.5 | 68.8 | 0.186 | 0.255 |
| Burkholderia thailandensis E254 | 68.9 | 68.7 | 0.188 | 0.255 |
| Burkholderia thailandensis strain 2003015869 | 68.8 | 68.6 | 0.188 | 0.250 |
