# Supplementary material for: Evolution of bopA Gene in Burkholderia: A Case of Convergent Evolution as a Mechanism for Bacterial Autophagy Evasion
Source: Biomed Res Int. 2016 Nov 27;2016:6745028. doi: 10.1155/2016/6745028 (PMC5149610; doi:10.1155/2016/6745028)
Supplement: Supplementary file 1 — The GC and CAI values for the gene BopA and the genome that BopA locates, respectively. [file 6745028.f1.zip › Table S2 accession numbers.docx]

Accession numbers of *Burkholderia* genomic sequences and *bopA* gene sequences used in this study

| Strain name | Burkholderia genome | BopA gene |
| --- | --- | --- |
| Burkholderia mallei ATCC 23344 | GCA_000011705.1 | CP000011.2 |
| Burkholderia mallei NCTC 10229 | GCA_000015605.1 | CP000545.1 |
| Burkholderia mallei NCTC 10247 | GCA_000015625.1 | CP000547.1 |
| Burkholderia mallei strain FMH 23344 | GCA_000755785.1 | CP009147.1 |
| Burkholderia mallei strain 6 | GCA_000755845.1 | CP008710.1 |
| Burkholderia mallei strain 23344 | GCA_000755865.1 | CP008705.1 |
| Burkholderia mallei strain BMQ | GCA_000755885.1 | CP008722.1 |
| Burkholderia mallei strain 2000031063 | GCA_000756025.2 | CP008731.2 |
| Burkholderia mallei strain 092700E | GCA_000762285.1 | CP007801.1 |
| Burkholderia thailandensis E264 | GCA_000012365.1 | CP000085.1 |
| Burkholderia thailandensis MSMB121 | GCA_000385525.1 | CP004096.1 |
| Burkholderia thailandensis H0587 | GCA_000567905.1 | CP004090.1 |
| Burkholderia thailandensis 2002721723 | GCA_000567925.1 | CP004098.1 |
| Burkholderia thailandensis E444 | GCA_000567945.1 | CP004118.1 |
| Burkholderia thailandensis USAMRU Malaysia #20 | GCA_000706745.1 | CP004384.1 |
| Burkholderia thailandensis MSMB59 | GCA_000764595.1 | CP004386.1 |
| Burkholderia thailandensis E254 | GCA_000765375.1 | CP004382.1 |
| Burkholderia thailandensis strain 2003015869 | GCA_000808035.2 | CP008915.2 |
| Burkholderia pseudomallei strain K96243, | GCA_000011545.1 | BX571966.1 |
| Burkholderia pseudomallei 1710b | GCA_000012785.1 | CP000125.1 |
| Burkholderia pseudomallei 668 | GCA_000015905.1 | CP000571.1 |
| Burkholderia pseudomallei 1106a | GCA_000015925.1 | CP000573.1 |
| Burkholderia pseudomallei 1026b | GCA_000260515.1 | CP002834.1 |
| Burkholderia pseudomallei BPC006 | GCA_000294635.1 | CP003782.1 |
| Burkholderia pseudomallei MSHR305 | GCA_000439695.1 | CP006469.1 |
| Burkholderia pseudomallei NCTC 13179 | GCA_000494855.1 | CP003977.1 |
| Burkholderia pseudomallei NCTC 13178 | GCA_000511895.1 | CP004002.1 |
| Burkholderia pseudomallei NAU20B-16 | GCA_000511915.1 | CP004004.1 |
| Burkholderia pseudomallei MSHR511 | GCA_000520895.1 | CP004024.1 |
| Burkholderia pseudomallei MSHR146 | GCA_000521645.1 | CP004043.1 |
| Burkholderia pseudomallei MSHR520 | GCA_000583835.1 | CP004369.1 |
| Burkholderia pseudomallei strain BDP | GCA_000755765.1 | CP009210.1 |
| Burkholderia pseudomallei strain BSR | GCA_000755825.1 | CP009127.1 |
| Burkholderia pseudomallei HBPUB10303a | GCA_000755905.1 | CP008893.1 |
| Burkholderia pseudomallei HBPUB10134a | GCA_000755925.1 | CP008912.1 |
| Burkholderia pseudomallei MSHR5858 | GCA_000755945.1 | CP008891.1 |
| Burkholderia pseudomallei MSHR5848 | GCA_000755965.1 | CP008910.1 |
| Burkholderia pseudomallei MSHR5855 | GCA_000756065.1 | CP008783.1 |
| Burkholderia pseudomallei strain BGR | GCA_000756085.1 | CP008835.1 |
| Burkholderia pseudomallei strain Mahidol-1106a | GCA_000756125.1 | CP008782.1 |
| Burkholderia pseudomallei strain 1106a | GCA_000756145.1 | CP008759.1 |
| Burkholderia pseudomallei strain MSHR1655 | GCA_000756165.1 | CP008779.1 |
| Burkholderia pseudomallei 576 | GCA_000756185.1 | CP008778.1 |
| Burkholderia pseudomallei NAU35A-3 | GCA_000764575.1 | CP004378.1 |
| Burkholderia pseudomallei MSHR62 | GCA_000770395.1 | CP009234.1 |
| Burkholderia pseudomallei B03 | GCA_000770455.1 | CP009150.1 |
| Burkholderia pseudomallei TSV 48 | GCA_000770495.1 | CP009160.1 |
| Burkholderia pseudomallei K42 | GCA_000770515.1 | CP009163.1 |
| Burkholderia pseudomallei A79A | GCA_000770535.1 | CP009164.1 |
| Burkholderia pseudomallei BP_3921g, | GCA_000953095.1 | LK936443.1 |
| Burkholderia pseudomallei strain vgh07 | GCA_000954175.1 | CP010974.1 |
